# Effects of COVID-19 Pandemic and Post-Vaccination Period on Gastroenterology Practice in Turkey

**DOI:** 10.5152/tjg.2022.22093

**Published:** 2023-01-01

**Authors:** Zeynep Gök Sargın, İbrahimhalil Düşünceli, Ümüt Çelik

**Affiliations:** Department of Gastroenterology and Hepatology, Zonguldak Bülent Ecevit University Faculty of Medicine, Zonguldak, Turkey

**Keywords:** COVID-19, COVID-PCR, endoscopic procedure, gastroenterologist, vaccination period

## Abstract

**Background:**

The sudden and intense burden due to the novel coronavirus (coronavirus disease 2019) pandemic has changed the priority of many health services. The highly contagious new variants and vaccination services have greatly impacted the daily practice of gastroenterologists. In the present study, we tried to evaluate the change in the daily practice of Turkish gastroenterologists in the coronavirus disease 2019 pandemic and the post-vaccination periods.

**Methods:**

A questionnaire was prepared using Google forms and sent online to 214 gastroenterologists registered with the Turkish Gastroenterology Association.

**Results:**

Approximately half of the gastroenterologists moved their endoscopy unit or gastroenterology clinic to another location in the hospital during the pandemic and about one-third of the endoscopy units were completely closed. About 56% of the respondents reported the interruption of endoscopic procedures and inpatient services. In total, 85% of gastroenterologists thought that follow-up and treatment of chronic patients were disrupted due to patients obtaining their medicine directly from pharmacies. After the first case in Turkey, 74% of gastroenterologists worked in coronavirus disease 2019 patient care services, 28% of gastroenterologists were infected with coronavirus disease 2019, and 3% believed they had a cross-infected patient(s). A total of 78% of gastroenterologists reported that patient management became difficult due to the change in the priority of other departments, and 60% of gastroenterologists confirmed that they experienced a decrease in income. In the post-vaccination period, 70% of gastroenterologists returned to their pre-pandemic working schedule and 31% noticed an increase in endoscopic cancer detection.

**Conclusion:**

Prolongation of the pandemic has seriously damaged the practice of gastroenterology and multidisciplinary patient management.

**Main Points**

- A nationwide online survey was conducted on 147 gastroenterologists in Turkey to assess their daily practice in the coronavirus disease 2019 (COVID-19) pandemic and the normalization process.
- Approximately half of the gastroenterologists moved their endoscopy unit or gastroenterology clinic to another location in the hospital during the pandemic, and about one-third of the endoscopy units were completely closed. A total of 74% of gastroenterologists worked in COVID-19 patient care services and 56% of respondents reported the interruption of endoscopic procedures and inpatient services.
- After vaccination, 71% of gastroenterologists returned to their pre-pandemic working schedule. However, Turkish gastroenterologists are still worried after vaccination and are careful about precautions.
- In the normalization period, 31% of gastroenterologists noticed an increase in endoscopic cancer detection.

## Introduction

Coronavirus disease 2019 (COVID-19) is a highly contagious and infectious viral disease caused by the severe acute ­respiratory syndrome coronavirus-2 (SARS-CoV-2).^1^ Since the first case of infection related to COVID-19 was first reported in December 2019 in Wuhan, Hubei Province, China, the infection has spread to more than 216 countries and regions, ultimately, the World Health Organization declared a pandemic on March 11, 2020.^2^ The primary mode of transmission of the virus is airborne droplets, though spread via aerosolized viral particles can also occur.^3^ It has been reported that endoscopy is an aerosol-generating procedure,^4^ and positive air insufflation during endoscopic procedures may pose a risk of aerosol generation and increase the risk of SARS-CoV-2 transmission. Therefore, gastroenterologists and endoscopy nurses are at risk during the COVID-19 pandemic through direct contact or aerosol droplets in endoscopic procedures. Guidelines for endoscopic procedures suggest triage of patients with suspected or confirmed COVID-19 before endoscopy, postponing elective endoscopies, and endoscopies should be performed in a negative pressure room in suspected or confirmed cases of COVID-19.^5,6^ However, some studies indicate that the risk is not high for health professionals and can be reduced with personal protective equipment (PPE).^7^ In this process, the consequences of postponing endoscopic procedures and the interruption of the follow-up of chronic patients, and whether the pandemic has caused financial and psychological losses for gastroenterologists are concerning. In addition, the new mutant peaks that emerged during the post-vaccine normalization period caused the pandemic to become inextricable and from time to time may lead healthcare professionals to despair about the sustainability of this process. The first COVID-19 case in Turkey was seen on March 11, 2020, and the first COVID-19 vaccine was administered on January 13, 2021. Therefore, there is very little data on the change in the practice of gastroenterology in these dynamic processes. In this study, we aimed to evaluate the changes in gastroenterology practice and the tolerance of gastroenterologists during the pandemic and the normalization periods.

## MATERIAL AND Methods

A questionnaire-based online survey was prepared using Google forms and sent to 214 gastroenterologists, including 77 specialists, 75 fellows, and 62 academicians in the field of gastroenterology (20 professors and 42 associate professors) registered with the Turkish Gastroenterology Association in Turkey. A total of 20 questionnaires were given to all participants, who were also clinicians, covering changes in the daily practice of gastroenterologists during the first wave of the pandemic and the post-vaccination normalization period. The details of the questionnaire are described in Supplementary Material 1. Two reminders were sent for 1 week period to the participants. Study participants were not given any gifts or payments; the questionnaire was answered voluntarily. This survey was conducted after 4 doses of vaccine were administered to health workers in Turkey. The survey was started on January 21, 2022, and the responses were collected by the end of January 2022. Permission from the Turkish National Ministry of Health and approval from Zonguldak Bülent Ecevit University Faculty of Medicine Non-Interventional Clinical Research Ethics Committee were obtained for the study (protocol number: 2022/01 approval date: January 12, 2022). The study protocol conforms to the ethical guidelines of the 1964 Declaration of Helsinki. No special statistical analysis was used. Descriptive data are presented either as means or medians for continuous variables; frequencies and percentages are reported for categorical variables.

## Results

A total of 214 participants were sent a questionnaire and 147 participants (69%) answered the questionnaire. When the subgroups of those who answered the questionnaire were evaluated, 42 of 75 fellows (56%), 12 of 20 professors (60%), 30 of 62 associate professors (71%), and 63 of 77 specialists (81%) answered the questionnaire. About 60% of the participants were between the ages of 30 and 40. Most of the participants were specialists (43%). Nearly 85% of the participants were working in a university hospital, training and research hospital, or state hospital (Table 1).

**Figure 1. f1-tjg-34-1-13:**
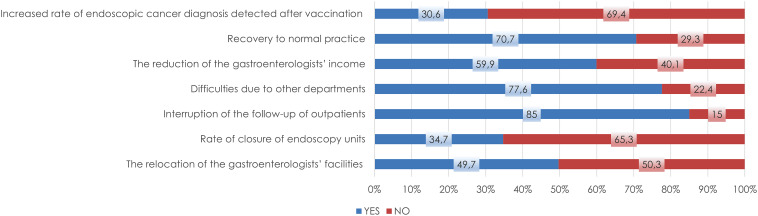
The effect of the pandemic on the practice of gastroenterology.

**Figure 2.**
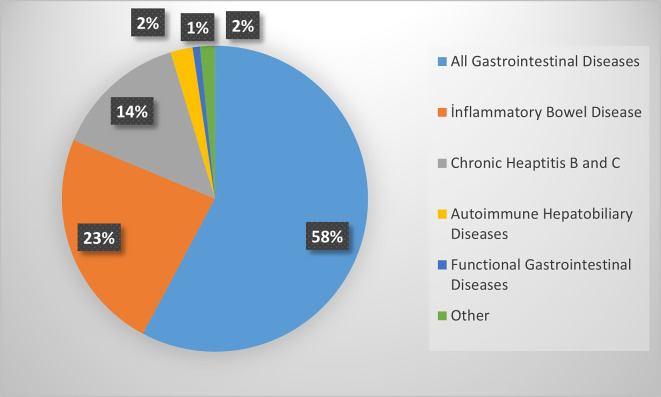
Chronic diseases, the management of which is considered to be the most disrupted.

Approximately half of the gastroenterologists (73) moved their endoscopy unit or gastroenterology clinic to another location in the hospital during the pandemic [Fig f1-tjg-34-1-13]and about one-third of (52) the endoscopy units were completely closed (Figure 1). A total of 56% (76) of the respondents reported the interruption of endoscopic procedures and inpatient services, and 85% (126) of gastroenterologists thought that follow-up and treatment of chronic patients were disrupted due to patients obtaining their medicine directly from pharmacies without visiting the hospital (Figure 1). Inflammatory bowel disease and chronic viral hepatitis compromised most of these patients (Figure 2). After the first case in Turkey, 74% (109) of gastroenterologists worked in COVID-19 patient care services; 41 (28%) gastroenterologists were infected with COVID-19, and 5 (3%) gastroenterologists believed they had a cross-infected patient(s).

**Figure 3.**
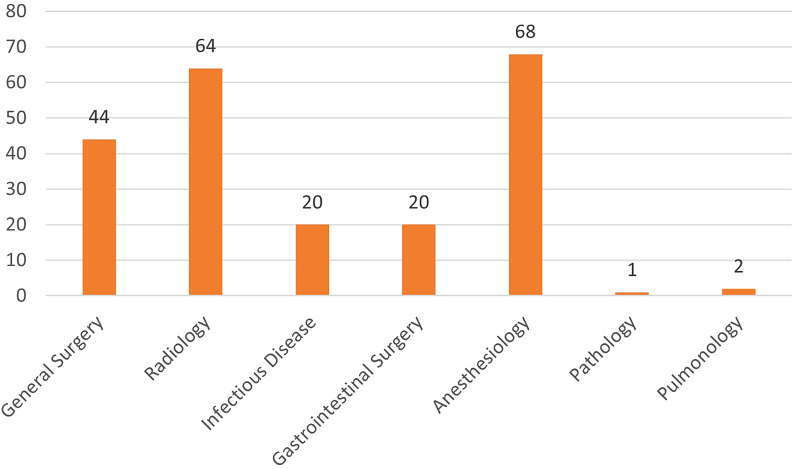
Other departments that disrupt patient management.

In total, 78% (115) of gastroenterologists reported that patient management became difficult due to the change in priority of other departments (delay in radiological examinations, biopsy, surgical procedures, etc.) (Figure 1). They reported that the specialties that mostly interrupted patient management were anesthesia (60%, 68), general surgery (39%, 44), and radiology (57%, 64) (Figure 3).

Throughout the pandemic, 60% (89) of gastroenterologists confirmed that they experienced a decrease in income (Figure 1) and 7% (10) of them reported receiving psychological support. Before vaccination, 77% (114) of gastroenterologists required patients to undergo a COVID-polymerase chain reaction (PCR) test before endoscopic procedures, and after vaccination, 73% (108) required patients to undergo PCR testing. After vaccination, 71% (105) of gastroenterologists returned to their pre-pandemic working schedule (Figure 1), however, 69% (102) continued to use PPE or additional precautions other than wearing a surgical mask during endoscopic procedures and after vaccination started (approximately 1 year after the first case), 31% (45) of gastroenterologists noticed an increase in endoscopic cancer detection (Figure 1).

## Discussion

In the present study, it was observed that the practice of gastroenterology was significantly affected by the pandemic. The change in the priority of health care services has significantly disrupted routine endoscopic procedures, outpatient, and inpatient services. The number of screening or follow-up endoscopies and therapeutic endoscopies decreased significantly during the first wave of the pandemic, as it was vigorously recommended to postpone or cancel non-urgent endoscopies to prevent the spread of SARS-CoV-2 by the health workers. Our findings are in line with an article analyzing the training of gastroenterology fellows in the COVID-19 pandemic; it was emphasized that 91.7% of standard diagnostic endoscopic procedures, 57.2% of standard therapeutic procedures, and 67.7% of advanced endoscopic procedures decreased in the first 7 months of the pandemic evaluated in that study.^8^ But this finding may not reflect the practice of all of the gastroenterologists; also, in our study, the majority of the participants are gastroenterology specialists. In a survey-based study conducted in Asia, with the first wave of the pandemic, diagnostic endoscopies decreased significantly compared to the pre-pandemic period.^9^ Similarly, in a study evaluating the effects of the COVID-19 pandemic on the endoscopy practice in China, it was emphasized that the number of endoscopies decreased by half during the pandemic.^10^

Our study showed that half of the gastroenterologists moved their endoscopy unit or gastroenterology clinic to another location in the hospital during the pandemic, and about one-third of the endoscopy units were completely closed. Considering that most of our participants work in public institutions, this result is within the expected rates. From Northern Italy, Repici et al^11^ reported that about 66% of endoscopy units and endoscopists have moved to other hospital departments to assist COVID-positive patient care services. A national survey reported that with the first wave of the pandemic, 55% of gastroenterology clinics in North America were partially closed and 21% were completely closed.^12^

The COVID-19 pandemic had a substantial impact not only on the practice of gastrointestinal endoscopy but also on the medical treatment for gastrointestinal diseases. During the pandemic period, patients obtained their medicines directly from pharmacies without coming to outpatient clinics as part of the Turkish ministry of health measures. According to our survey, most of the gastroenterologists (85%) thought that the follow-up and treatment of chronic patients were disrupted, the most affected were inflammatory bowel disease and chronic viral hepatitis. It was found that functional dyspepsia and irritable bowel syndrome were exacerbated by the COVID-19 pandemic in Asia study.^9^

It suggests that delaying elective endoscopic procedures and reducing the number of procedures may also impair the education of fellows who constitute 29% of our study participants. In a study conducted in Turkey among gastroenterology fellows, 56% of them reported a decrease in independently performed endoscopic procedures.^8^ Similarly, in a study investigating the effects of the COVID19 pandemic on general surgery training in Turkey, there was a significant decrease in the number of elective and emergency surgeries, and therefore, they thought that general surgery residency training might be affected.^13^

In this survey, Turkish gastroenterologists reported the departments that most disrupted the management of their patients were anesthesia, general surgery, and radiology. Because the virus contained in the pneumoperitoneum is released in the operating room in the form of aerosols, surgeons have proposed to minimize the risk of exposure to the virus even in emergency operations by involving a minimum number of medical personnel and minimizing exposure to the operating room.^14^ Therefore, elective surgeries have been postponed except for emergency surgeries. In addition, gastroenterologists may have encountered difficulties as anesthetists had to delay endoscopic procedures as they were redeployed to intensive care units. Elective patient imaging or interventional radiological procedures may have been disrupted due to overload in COVID-19-related chest CT and chest radiographs.

Strikingly, in this study, approximately 30% of the participants noticed an increase in the diagnosis of endoscopic cancer during the transition to normalization. This increase is thought to be due to a delay in diagnostic endoscopies during the pandemic. The time from the first case to appear in our country until the start of vaccination is about 1 year, and we think that cancer cases are more common in the normalization process due to the delay in this 1 year. In a multicenter study, in accordance with our results, 39% of respondents reported cases of delayed diagnosis of cancer due to delayed endoscopic procedures.^9^ In a multicenter study on cytopathological samples from 23 countries, during the quarantine period of the COVID-19 pandemic, it is predicted that the total number of cytology samples, regardless of the anatomical region or sample type, will decrease significantly and the cancer rates will increase prospectively. Prospective monitoring of the impact of delays in accessing healthcare during the quarantine period is recommended.^15^ Similarly, an online survey of cytopathology laboratories in 24 Asia-Pacific countries to investigate the impact of restrictive measures on healthcare access, use of PPE, changes in cytology workflow, showed that the COVID-19 pandemic resulted in a significant reduction in the number of cytology specimens examined.^16^

We also observed that approximately 60% of gastroenterologists reported a reduction in income and 74% of gastroenterologists worked in COVID-19 patient care services in the pandemic. It was thought that financial concerns and taking a role in COVID-19 patient care may impair the motivation of physicians but only 7% received psychological support. Turkish oncologists also reported that they experienced similar losses during the pandemic process. It was also emphasized that the protracted pandemic undermined oncology practice through the loss of motivation of oncologists and incomplete multidisciplinary patient management.^17^ In a survey study investigating the impact of the COVID-19 pandemic on gastroenterologists in Southeast Asia, it was stated that the majority of gastroenterologists were under stress and experienced burnout syndrome in a pandemic, but more than half were unaware of psychological support.^18^

According to this survey, approximately 70% of the gastroenterologists returned to their previous working order after vaccination (approximately 1 year later after the first case in Turkey), and we believe that this rate will increase with the rising immunity and decreasing incidence of infections and hospitalizations. The study conducted in Asian countries emphasized that the volume of screening or follow-up endoscopy did not fully return in most Asian countries after the first wave.^9^ However, a significant portion of endoscopic procedures in Thailand resumed after the first wave of the pandemic; this has been associated with strict quarantine and meticulous use of PPE in the pandemic.^19^

In the present study, before vaccination, 77% of gastroenterologists required patients to undergo a PCR test before endoscopic procedures. On the other hand, Otani et al^9^ stated that the COVID-PCR test was performed before 40% of follow-up and screening endoscopies and 30% of emergency endoscopies, and these results were lower than our results. In addition, only 15% of African survey participants used PCR testing as a pre-screening measure in the pandemic.^20^ It seems that the requirement for PCR testing from patients has not changed much between the early period of the pandemic and the post-vaccination normalization period. Furthermore, about 70% of participants reported that they took additional precautions other than a surgical mask in the post-vaccination period. These results show that Turkish gastroenterologists are still worried after vaccination and are careful about precautions. A total of 72% of Turkish gastroenterologists were not infected with COVID-19. This may be because patients are still routinely tested for COVID-19 before endoscopic procedures during the pandemic and normalization period.

Since most of our survey respondents are specialists or academic staff in the field of gastroenterology, we think that this study reflects the views of Turkish gastroenterologists about the early period of the COVID-19 pandemic and the post-vaccine normalization period. Furthermore, we have not found a study in the literature evaluating the change in the practice of gastroenterology in both the pandemic and the post-vaccination (normalization) period.

The most important limitation of our study is that it is a cross-sectional study nationwide and was conducted only on gastroenterologists in Turkey. Therefore, these results may not be generalized to other countries. Another limitation is that some of the questions were based on subjective data. In addition, we could not ask more detailed questions so as not to reduce the participants’ response rate (for example, we accepted that they were all fully vaccinated because the participants were health workers, we did not ask how many doses they had been vaccinated).

## Conclusion

The prolonged pandemic has seriously damaged the practice of gastroenterology and multidisciplinary patient management. During the normalization period, gastroenterologists are still careful about precautions in endoscopic procedures. One-third of gastroenterologists noticed an increase in endoscopic cancer detection rates, and follow-up studies are needed to evaluate the status of the change in the COVID-19 pandemic.

## Figures and Tables

**Table 1.** Demographic Characteristics of the Participants

	n	%
Age		
30-35	32	21
36-40	57	38
41-45	29	20
46-50	19	13
51-55	10	7
56-60	1	1
Title		
Fellow	42	29
Specialist	63	43
Associate professor	30	20
Professor	12	8
Working hospital		
University hospital	70	46
State hospital	20	14
Training and research hospital	38	26
Private hospital	20	14

## Supplementary material 1

### Study Questionnaire Questions and Responses

1. How old are you? (147 responses)
  - 30-35 years, 32 (21%)
  - 36 -40 years, 57 (38%)
  - 41-45 years, 29 (20%)
  - 46-50 years, 19 (13%)
  - 51-55 years, 10 (7%)
  - 56-60 years, 1 (1%)
  - 61 years and above, 0
2. What is the position you are working in as a gastroenterologist? (147 responses)
  - Fellow, 42 (29%)
  - Specialist, 63 (43%)
  - Associate professor, 30 (20%)
  - Professor, 12 (8%)
3. What is the institution you are working at? (147 responses)
  - State hospital, 20 (14%)
  - University hospital, 70 (46%)
  - Training and research hospital, 38 (26%)
  - Private hospital, 20 (14%)
4. Has your endoscopy unit, inpatient clinic, or outpatient clinic been relocated within the hospital after the COVID-19 pandemic started in our country? (147 responses)
  - Yes, 73 (50%)
  - No, 74 (50%)
5. Has the endoscopy unit that you worked in closed (converted to another use) after the COVID-19 pandemic started in our country? (147 responses)
  - Yes, 52 (35%)
  - No, 95 (65%)
6. Have you been in the following pandemic care services after the COVID-19 pandemic started in our country? (You can choose multiple options) (147 responses)
  - COVID-19 outpatient clinic, 50 (34%)
  - COVID-19 inpatient clinic, 82 (56%)
  - COVID-19 intensive care unit, 32 (22%)
  - Emergency service, 25 (17%)
  - No, I haven’t been found, 38 (26%)
7. Have your endoscopic procedures and inpatient services been disrupted? (135 responses)
  - Yes, 76 (56%)
  - No, 59 (44%)
8. Have you had a COVID-19 infection? (147 responses)
  - Yes, 41 (28%)
  - No, 105 (72%)
9. Have you caused cross-infection in your patients while working in COVID-19 pandemic services? (147 responses)
  - Yes, 5 (3%)
  - No, 143 (97%)
10. Has your patient management become difficult due to disruptions in other departments during the COVID-19 pandemic? (Delay in radiological examinations, inability to perform surgery, etc.) (147 responses)
  - Yes, 115 (78%)
  - No, 32 (22%)
11. If your answer to the above question is yes, which branches have made patient management more difficult because of them? (You can choose multiple options) (113 responses)
  - General surgery, 44 (30%)
  - Radiology - interventional radiology, 64 (43%)
  - Gastroenterological surgery, 20 (14%)
  - Infectious diseases, 20 (14%)
  - Anesthesia and reanimation, 68 (46%)
  - Pathology,1 (1%)
  - Pulmonology, 2 (1%)
12. Have you suffered a loss of income during the COVID-19 pandemic? (147 responses)
  - Yes, 88 (60%)
  - No, 59 (40%)
13. Have you received psychological support during the COVID-19 pandemic? (147 responses)
  - Yes, 10 (7%)
  - No, 137 (93%)
14. Did you require patients to undergo a PCR test before endoscopic procedures in the COVID-19 pandemic before the ­vaccination period? (147 responses)
  - Yes, 114 (77%)
  - No, 33 (23%)
15. Do you still require patients to undergo a COVID-PCR test before endoscopic procedures in the post-vaccination period? (147 responses)
  - Yes, 108 (73%)
  - No, 39 (27%)
16. Did you switch to the same working order in the post-­vaccination period as before the COVID-19 pandemic? (147 responses)
  - Yes, 104 (71%)
  - No, 43 (29%)
17. Do you take extra precautions other than wearing a surgical mask and gown during endoscopic procedures in the post-­vaccine period? (You can choose multiple options) (147 responses)
  - No, 46 (31%)
  - 2 or more surgical masks, 23 (16%)
  - N95, FFP3 mask, 77 (52%)
  - Goggles or face shield, 37 (25%)
  - Double-gowning or double-gloving, 29 (20%)
  - Increasing the ventilation and disinfection conditions of the endoscopic procedure room, 23 (16%)
  - Using an endoscopic procedure cabinet, 2 (1%)
18. Did you notice an increase in the number of cancers with ­endoscopic diagnosis after you switched to normal working order after vaccination? (147 responses)
  - Yes, 45 (31%)
  - No, 102 (69%)
19. During the pandemic period, patients received their medicines directly from the pharmacy without coming to the outpatient clinic as part of the Turkish ministry of health measures. Do you think that the management of these patients is disrupted? (147 participants answered)
  - Yes, 126 (85%)
  - No, 21 (15%)
20. If your answer to the above question is yes, which patients do you think are more likely to have management problems? (128 participants answered)
  - Inflammatory bowel disease, 30 (23%)
  - Chronic hepatitis B or chronic hepatitis C, 18 (14%)
  - Autoimmune hepatobiliary diseases, 3 (2%)
  - Functional gastrointestinal diseases, 1 (1%)
  - All of them, 74 (58%)
  - Other diseases, 2 (2%)
